# Acknowledgment to Reviewers of *Tropical Medicine and Infectious Disease* in 2020

**DOI:** 10.3390/tropicalmed6010014

**Published:** 2021-01-25

**Authors:** Tropical Medicine Infectious Disease Editorial Office

**Affiliations:** MDPI AG, St. Alban-Anlage 66, 4052 Basel, Switzerland; tropicalmed@mdpi.com

Peer review is the driving force of journal development, and reviewers are gatekeepers who ensure that *Tropical Medicine and Infectious Disease* maintains its standards for the high quality of its published papers. Thanks to the cooperation of our reviewers, in 2020, the median time to first decision was 18 days and the median time to publication was 44 days. The editors would like to express their sincere gratitude to the following reviewers for their precious time and dedication, regardless of whether the papers were finally published:
Abd El Wahed, AhmedHorton, Daniel L.Acquatella, HarryHouzé, SandrineAdégbitè, Bayodé RoméoHsu, LewisAfolabi, MuhammedHuang, G. Khai LinAgopian, A. J.Huang, Yi-WenAkdesir, EzgiHudda, NeelakshiAlfson, KendraHuits, RalphAlger, JackelineHullsiek, Kathy H.Allegaert, KarelHung, Chin-ChuanALLEN, KELLYHussain, HamidahAlsford, SamInayathullah, MohammedAnderson, JohnIngle, DanielleAnstead, Gregory M.Inocencio Da Luz, RaquelAssunção-Miranda, IranaiaIsabel Veiga, MariaAuer, ChristianIyer, JananiAuguste, Albert J.Jackson, AlanAugustinos, AntoniosJakovljevic, MihajloAye, KhinJankowiak, WilliamAyhan, NazlıJanmaat, AlidaBanadyga, LoganJimenez-Sousa, Maria AngelesBara, JeffreyJohnson, NicholasBarbić, LjuboJones, Kathryn MarieBarreiro, PabloJones, MalcolmBartholomeeusen, KoenJurke, AnnetteBaryeh, KwakuJutla, AntarpreetBasco, LeonardoKaca, WiesławBattah, BasemKadima Ebeja, AugustinBaud, DavidKandil, Omnia M.Baymakova, MagdalenaKarganova, GalinaBelo, SilvanaKarolyi, MarioBeloukas, ApostolosKatuchova, JanaBeloukas, ApostolosKatzenstein, TerezeBenach, JorgeKawano, Daniel FábioBerhanu, Rebecca (Ribka)Kelly, PatrickBernardo, UmbertoKhubchandani, JagdishBerrilli, FedericaKimball, Ann MarieBhattacharyya, TapanKoekemoer, LizetteBiritwum, Nana KwadwoKondru, NaveenBisser, SylvieKones, RichardBlacksell, StuartKontsevaya, IrinaBlasdell, KimKorou, Laskarina-MariaBlicharski, TomaszKrammer, FlorianBlum, JohannesKumar, NirbhayBordin, LucianaKunz, JoachimBourque, Daniel LeeKuo, Chi-ChienBradbury, RichardKupz, AndreasBragazzi, Nicola LuigiKwan, JenniferBrandenberger, JuliaLangereis, Jeroen D.Brault, AaronLankester, FelixBriggs, DeborahLazuardi, LutfanBrown, Catherine MLeaver, David J.Brown, MarkLecollinet, SylvieBruisten, SylviaLee, Sang-SupBurns, TheresaLi, ChunfengCalistri, PaoloLiegner, KennethCampbell, JonathonLlanes, AlejandroCano, JorgeLodh, NilanjanCarr, Jillian M.Loureiro, Livia O.Caruso, GerardoLoVerde, PhilCarvalho, Danilo OliveiraLucchese, LauraCastelli, FrancescoLütticken, RudolfCastro-Escarpulli, GracielaMaatouk, IsmaelCatzeflis, FrançoisMacdonald, MichaelCazelles, BernardMacLeod, AnnetteChalla, DilipMaiega, HamidouChan, Hsun-YuMajumdar, SumanChandran, ArunaMalchiodi, Emilio L.Chang, Tsung-HsienManuel Giorgi, FedericoChang, Wen NengMarcovici, GenoChao, Chien-ChungMari, LorenzoChatelain, EricMaria, Naomi I.CHAUHAN, NIKHILMartínez, MartaChaves, Luis FernandoMartins, Ivo C.Chávez-Tapia, Norberto C.Masuzawa, ToshiyukiChen, Ya-LeiMateus-Pinilla, NohraCheng, QuMatovu, EnockChisari, EmanueleMattapallil, JosephChng, Nai RuiMattiuzzo, GiadaChoudhary, Kumari SonalMauk, Michael G.Christofferson, RebeccaMavian, CarlaChuang, Hung-YiMaze, Michael J.Ciccacci, FaustoMcBride, Jere W.Claborn, DavidMcBride, JohnClark, Nicholas J.Mendenhall, IanColquhoun, SamanthaMendoza-Cano, OliverColton, Jonathan S.Metz, Stefan W.Corlateanu, AlexandruMichels, PaulCoubally, JeanMiller, AdrianCouderc, ThérèseMills, KerryCourtin, FabriceMin, ZawCourtioux, BertrandMiron, Isidro JuanCoyle, Christina M.Molina, IsraelCreswell, JacobMoniuszko, HannaCrooks, GeorgeMorales-Morales, DavidCunha-Melo, JoséMorand, SergeCuric, GoranMora-Peris, BorjaCwynar, PrzemyslawMorozova, OlgaDance, David A. B.Morrone, AldoDasch, Gregory A.Mourad, AhmadDe Diego Cordero, RocíoMukherjee, RajDe Koning, HarryMulkey, SarahDe Oliveira, Everton FalcãoMüller, JanisDe Ory, FernandoMüller, UweDe Simone, Salvatore GiovanniMusch, MarkDeLuca, NickolasMutinelli, FrancoDiaz, James HenryNaish, SuchithraDiefenbach-Elstob, TanyaNajdenski, HristoDiel, RolandNavas, JesúsDkhar, Hedwin KitdorlangNeema, StellaDonald, Claire L.Nemeș, Roxana MariaDoucoure, SouleymaneNeumayr, AndreasDressler, FrankNg, CarolineDriscoll, TimothyNgatu, NlanduDuCros, PhilippNgwa, Che JuliusEcheverria, LuisNishizono, AkiraEckman, James R.Nolan, MelissaEI Ridi, RashikaNucera, EleonoraEisler, MarkNunziata, AngelinaEksioglu, SandraOh, Kyung HyunElbadry, Maha AOliver, JoAnneElmouki, IliasOnozaki, IkushiElson, LynneOoi, Eng EongFairlamb, AlanOsborne, JaneFan, Chia-KwungOuedraogo, Andre LinFei, Andrew Chang-YoungPadureanu, VladFernandes, Luis Guilherme VirgílioPaes, Marciano VianaFolkvardsen, Dorte BekPalmer, JenniferFonseca, KevinPaloque, LucieFraga, HugoPandey, AbhishekFranco-Molina, Moises ArmidesPanwar, PriyankaFranco-Paredes, CarlosParis, LucFrant, MaciejParvez, MohammadFrei, ThomasPatel, MoosaFrentiu, FrancescaPaul, RichardFreuling, ConradPaulson, SallyFujita, KoheiPelfrene, EricFurin, Jennifer J.Pépin, MichelGabrieli, PaoloPerez, Lark J.Galán-Puchades, María TeresaPeriago, Maria VictoriaGalvão, CleberPetersen, EskildGancheva, Galya IvanovaPettersson, JohnGarcia Nicolas, ObdulioPeyton, David H.García-Bujalance, SilviaPezzoni, GiuliaGarcía-R, Juan CarlosPhalen, DavidGaultier, Cyril R.Phulera, SwastikGavriilaki, EleniPiñeiro, LuisGerlich, WolframPlucinski, Mateusz M.Giacani, LorenzoPons-Duran, ClaraGibson, AndyPop, Tudor LucianGibson, WendyPulido, OlgaGislason, MayaQu, NingGoldsmid, JohnRajeev, SreekumariGómez-Barrena, EnriqueRamasamy, RanjanGonzález Cappa, Stella MarisRani Banik, GouriGonzález, Florenci V.Rashid, HarunorGordon, Catherine A.Reeves, Lawrence E.Grab, DennisRequena, Jose MGraham, StephenReves, RandallGrant, MatthewRiddick, Eric WellingtonGraves, StephenRiehle, MichaelGrippi, FrancescaRoberts, Drucilla J.Gross, AaronRobinson, MathewGryschek, Ronaldo Cesar BorgesRodgers, JeanGunesekera, AmilaRodrigues, RogérioGuo, Ming-LeiRomano, ClaudioGupta, HimanshuRonald Hope, KempeGustafson, PaulRondoni, GabrieleHamada, YohheiRoot, JeffHamarsheh, OmarRoque, André Luiz RodriguesHamel, RodolpheRosa, Bruce A.Hamilton, Clare M.Rosana Rocha, BarrosHampson, KatieRowe, ReginaHansen, DianaRuddraraju, Kasi ViswanatharajuHanson, KirstenRuktanonchai, Nick WarrenHarkin, Kenneth R.Salamon, DominikaHarries, Anthony D.Salje, JeanneHarrod, RobertSalomón, Oscar D.Harrus, ShimonSanchez Vargas, Luis AlbertoHattori, ToshioSanderson, PriscillaHavik, Philip J.Santillan Garcia, AzucenaHawman, DavidSapp, SarahHeffernan, MargaretSaraiva, Raúl G.Herchenröder, OttmarSarr, MoussaHerrick, Jesica AllynSato, Ana Paula SayuriHodgson, IanSaul, SirleHoff, Rodney


